# Genetic diversity of drug and multidrug-resistant *Mycobacterium tuberculosis* circulating in Veracruz, Mexico

**DOI:** 10.1371/journal.pone.0193626

**Published:** 2018-03-15

**Authors:** Daniela Munro-Rojas, Esdras Fernandez-Morales, José Zarrabal-Meza, Ma. Teresa Martínez-Cazares, Aurora Parissi-Crivelli, Javier Fuentes-Domínguez, Marie Nancy Séraphin, Michael Lauzardo, Jorge Alberto González-y-Merchand, Sandra Rivera-Gutierrez, Roberto Zenteno-Cuevas

**Affiliations:** 1 Instituto de Salud Pública, Universidad Veracruzana, Jalapa, Veracruz, México; 2 Programa de Doctorado en Ciencias de la Salud, Instituto de Ciencias de la Salud, Universidad Veracruzana, Veracruz, México; 3 Programa de Maestría en Ciencias de la Salud, Universidad Veracruzana, Veracruz, México; 4 Laboratorio Estatal de Salud Pública, Secretaria de Salud, Veracruz, México; 5 Programa Estatal de Micobacteriosis, Secretaria de Salud, Veracruz, México; 6 Division of Infectious Diseases and Global Medicine, College of Medicine, University of Florida, Gainesville, Florida, United States of America; 7 Escuela Nacional de Ciencia Biológicas, Instituto Politécnico Nacional, Ciudad de México, México; St. Petersburg Pasteur Institute, RUSSIAN FEDERATION

## Abstract

**Background:**

Mexico is one of the most important contributors of drug and multidrug-resistant tuberculosis in Latin America; however, knowledge of the genetic diversity of drug-resistant tuberculosis isolates is limited.

**Methods:**

In this study, the genetic structure of 112 *Mycobacterium tuberculosis* strains from the southeastern Mexico was determined by spoligotyping and 24-loci MIRU-VNTRs.

**Findings:**

The results show eight major lineages, the most of which was T1 (24%), followed by LAM (16%) and H (15%). A total of 29 (25%) isolates were identified as orphan. The most abundant SITs were SIT53/T1 and SIT42/LAM9 with 10 isolates each and SIT50/H3 with eight isolates. Fifty-two spoligotype patterns, twenty-seven clusters and ten clonal complexes were observed, demonstrating an important genetic diversity of drug and multidrug-resistant tuberculosis isolates in circulation and transmission level of these aggravated forms of tuberculosis. Being defined as orphan or as part of an orphan cluster, was a risk factor for multidrug resistant-tuberculosis (OR 2.5, IC 1.05–5.86 and OR 3.3, IC 1–11.03, respectively). Multiple correspondence analyses showed association of some clusters and SITs with specific geographical locations.

**Conclusions:**

Our study provides one of the most detailed description of the genetic structure of drug and multidrug-resistant tuberculosis strains in southeast Mexico, establishing for the first time a baseline of the genotypes observed in resistant isolates circulating, however further studies are required to better elucidate the genetic structure of tuberculosis in region and the factors that could be participating in their dispersion.

## Introduction

Over recent decades, the worldwide resurgence of tuberculosis (TB) has been a challenge for health institutions. According to the global TB report of the World Health Organization, in 2015, TB was responsible for the deaths of 1.5 million people and generated 10.4 million cases, of which 100,000 were drug resistant tuberculosis (DR-TB) with specific resistance against rifampicin-resistant (RR-TB), 480,000 presenting combined resistance to rifampin and isoniazid and considered as multidrug resistant (MDR-TB), and 7579 showed extreme drug resistance (XDR-TB) [1]. In 2015, only 125,000 (20%) of the 480,000 individuals with RR and MDR, and 7234 patients with XDR-TB were enrolled for treatment; however, successful outcomes were 83% for patients with TB (2014 cohort), 52% for MDR/RR-TB and 28% for XDR-TB. These figures clearly demonstrate the magnitude of the drug resistance phenomenon in TB, and its impact on the goal for eradication of TB by 2030[2].

The use of tools that allow characterization and genotypic analysis of TB, such as 24-locus mycobacterial interspersed repetitive unit-variable-number tandem-repeat (MIRU-VNTR) and spacer oligonucleotide typing (spoligotyping), allow an understanding of the dynamics and complexity of the population structure of *Mycobacterium tuberculosis* within a population [3,4]. These procedures allow identification of the different lineages in circulation within specific regions and their relationship with potential pathogenicity and virulence [5,6] Also, several reports from different geographic regions have described the levels of association with demographic, epidemiological and drug resistance characteristics [7–12].

According to the global TB report by the WHO, 22,869 new cases of TB were reported in 2016 in Mexico, with an incidence of 22/100,000 inhabitant and 610 cases of MDR-TB [1], and according to the Pan American Health Organization (PAHO) is placed as one of the five countries with more contributions of DR-TB and MDR-TB cases in Latin America [13]. However, there is limited data on the genotypic characteristics of the TB strains in circulation [14–16], including the drug and multidrug resistant isolates [14,17–20]. Moreover, there is limited data on the association between TB lineages in circulation and the clinical, epidemiological and drug resistant profiles of these isolates [18]. The aim of the present study is therefore to characterize the genetic diversity of DR and MDRTB strains isolated in Southeast Mexico and identify risk factors for infection with the specific drug-resistant lineages circulating in Veracruz, Mexico.

## Material and methods

### Study population and clinical sample isolation

Conducted between April 2013 and May 2015, this is a cross-sectional study in which 400 clinical sputum samples were taken from the same number of individuals suspected of having TB. The Public Health Laboratory of the Veracruz State Health Department collected all samples as part of the standard diagnostic procedure to confirm presence of TB and drug resistant. This laboratory provides the service of diagnosis of TB and also the susceptibility test against first line drugs, to the population of state of Veracruz; which is close to 7.5 million inhabitants, placed in an area close to 72,000 km^2^. Sputum samples were decontaminated using Petroff´s modified method [21] and primary isolation was achieved with Löwenstein-Jensen medium. Susceptibility testing for the first line drugs streptomycin (S), isoniazid (H), rifampin (R), ethambutol (E) and pyrazinamide (Z) was performed using the fluorometric method (MGIT 960 Becton-Dickinson)

**Fig 1:**
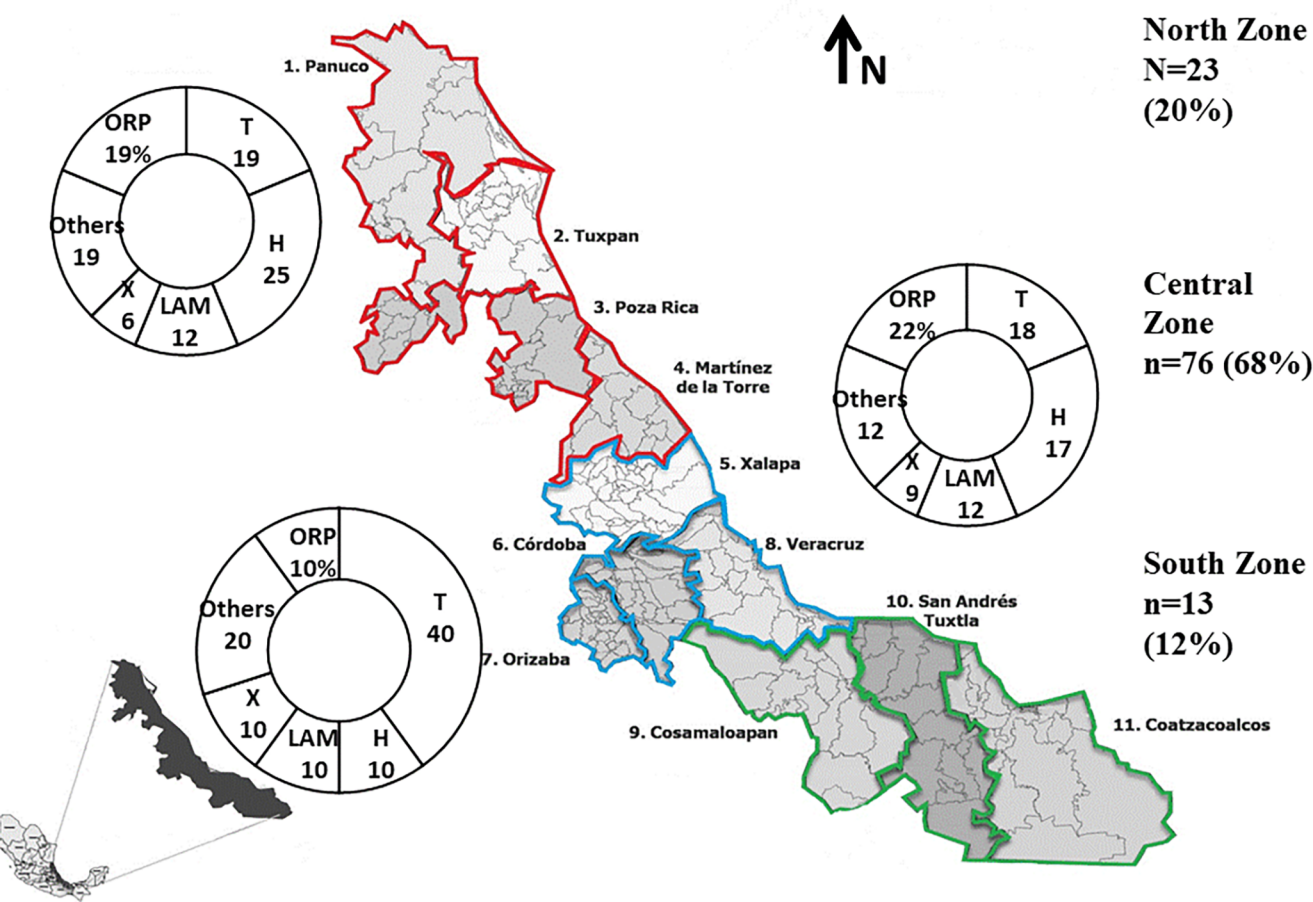
Distribution of the geographical zones, jurisdictions and lineages included in Veracruz, Mexico. North Zone (NZ): Panuco, Tuxpan, Poza Rica, Martínez de la Torre. Central Zone (CZ):Xalapa, Córdoba, Orizaba, Veracruz. South Zone (SZ): Cosamaloapan, San Andrés Tuxtla, Coatzacoalcos.

Variables such as age, gender, place of residence, type of diagnosis and treatment were recovered from the clinical files of each patient. Geographical characteristics were included considering two levels; (1) the jurisdiction or “district” where each isolate was collected, and (2) the geographical zone in which the health jurisdictions were located. Considering these three major zones were defined: North Zone including the following jurisdictions: 1 (Panuco), 2 (Tuxpan), 3 (Poza Rica) and 4 (Martinez de la Torre), Central Zone considering: 5 (Xalapa), 6 (Cordoba), 7 (Orizaba), 8 (Veracruz), and South Zone including: 9 (Cosamaloapan), 10 (San Andres Tuxtla) and 11 (Coatzacoalcos). (Fig 1)

#### DNA purification, 24-loci MIRU-VNTR and spoligotyping analysis

The DNA was extracted with a loop of cultured mycobacteria, following the recommendations of Van Soolingen et al.[22]. Concentration of the DNA was determined by spectrophotometry in a Nanodrop 1000 (Thermo Scientific). The 24-locus MIRU-VNTR typing was conducted following the recommendations of Supply et al. [4]. Each locus was amplified individually and PCR fragments were separated by electrophoresis using a 2% agarose gel. Fragment size was estimated by comparison with 100 bp DNA molecular weight ladders and independently verified by two separate individuals. The number of repeats at each locus was calculated by applying the corresponding conversion table [23].

Spoligotyping was carried out following standard techniques [3,24]. The direct repeat (DR) region was amplified with the oligonucleotides *DRa* (5´-GGTTTTGGGTCTGACGAC-3´ biotinylated) and *DRb* (5´-CCGAGAGGGGA CGGAAAC-3´). The biotinylated PCR products were hybridized to a membrane containing a set of 43 oligonucleotides corresponding to each spacer. DNA from *M*. *tuberculosis* H37Rv, *M*. *tuberculosis* CDC1551 and *M*. *bovis* BCG were used as controls. Hybridized PCR products were incubated with streptavidin peroxidase conjugate, and the membrane exposed to a chemiluminescence system, followed by exposure to X-ray film. The film was then developed using standard photochemical procedures (Amersham International, Buckinghamshire, United Kingdom). Spoligotype international type (SIT), and family assignment was performed using databases SITVIT3 and SPOLDB4 (http://www.pasteur-guadeloupe.fr/tb/bd_myco.html)[25,26].

#### Lineage assignment, cluster and clonal complex analysis

Genotypes were expressed as numerical codes representing the number of MIRU-VNTR for each locus. Lineages were individually identified using the similarity search module [27] of the database MIRU-VNTRplus (http://www.miru-vntrplus.org/MIRU/index.faces)[28].

The tree polar arrangement was realized by the FigTree program (Tree Figure Drawing Tool Version 1.4.3. http://tree.bio.ed.ac.uk/software/figtree), through Nexus file generated by algorithm UPGMA on database *MIRU-VNTRplus* considering data of spoligotyping patterns and 24 loci-MIRU- VNTR. The Hunter-Gaston discriminatory index (HGDI) was calculated as previously reported for 24 loci-MIRU-VNTR [29].

Clonal complexes were identified using the minimum spanning tree module in the database *MIRU-VNTRplus* [28], considering 24-MIRU-VNTR loci, with maximum differences within a clonal complex of five loci.

### Statistical analysis and association of epidemiological variables and genotypes

Data from the patients included in the study was analyzed using frequencies and Chi-square statistics with Yates´s correction and Fisher´s exact test. Association between the variables was determined based on the analysis of odds ratios, considering inclusion within a specific lineage, clusters or SITs and variables such as gender, age, geographical location (Jurisdictions or geographical zone) and drug-resistance considering a value of *p<0*.*05* to be significant. All calculations were performed using the software SPSS V.12. To further explore the relationship between characteristics such as gender, age, drug resistance profile, jurisdiction, clusters and SITs, we performed a multiple correspondence analysis[30]. This analysis is used to identify, within a small number of dimensions, the most significant deviations that are determined by a chi square function, which is known as the total inertia. Dimension 1 represents the largest deviation of independence from the data, and dimension 2 the second largest deviation, and so on in descending order. The more distant a value is from the origin of the category of response in a dimension, the greater its importance in interpreting this dimension. Clusters or categories of response that are very close within a point plot, and with their load in the same dimension, tend to indicate similarities between responses with moderate to high correlations [30].

### Ethical concerns

No physical interventions took place with the patients. All of the information collected was treated as confidential and written consent was obtained from each individual. The Ethics committees of the Public Health Institute at University of Veracruz oversaw and approved the ethical issues involved in this study, and no experimental animals were included.

## Results

### Population and clinical samples

During the period April 2013 to May 2015, 410 sputum samples from patients diagnosed with pulmonary TB were collected from the eleven health jurisdictions from Veracruz as described above. Of these, one hundred twelve (25%) isolates showed resistance to at least one first-line drug and were therefore included in the analysis.

Individuals bearing a drug resistant strain were predominantly male (79 individuals; 71%) with an average age of 44 ± 16.7 years. By age group, 18 (16%) of persons were between 18 and 24 years of age, 38 (34%) between 25–44 years and 56 (50%) over 45 years. A total of Ninety one (81%) individuals were diagnosed with TB for the first time, while 21 (15%) had had previous treatment.

### Characteristics and geographic distribution of drug resistant isolates

Twenty different drug resistance profiles were identified; 31 (28%) strains were mono-resistant (MR), 18 (16%) had resistance against any combination of two or more drugs, with exception of the combination rifampicin and isoniazid, were recognized as poly-resistant (PR), 62 (55%) were MDR and one (1%) was extreme drug-resistant. This last isolate was initially identified as MDR, but later confirmed as XDR by the National Institute of Epidemiological Reference, México.

Of the 31 MR isolates, eleven presented resistance to isoniazid (H), ten with resistance to Streptomycin, seven to rifampicin and three to pyrazinamide. No mono-resistance to ethambutol (E) was found. In the PR isolates, eight different profiles were identified. Of these, the highest proportion (9/18) was a combination of isoniazid and streptomycin resistance (HS). Among the MDR isolates, 8 different resistance profiles were observed and 21% (13/62) presented resistance to all first-line drugs (SHREZ).

According to their origin, 23 (20.5%) isolates came from the North Zone (NZ, including health jurisdictions 1–4), while seventy six (67.8%) were found at the Central Zone (CZ, including jurisdictions 5–8) and 13 (11.16%) came from the South Zone (SZ, including jurisdictions 9–11). Considering the MDR character of the TB isolates, the Central Zone provided the largest number of MDR isolates (45/76), followed by the NZ (13/23) and finally the SZ (4/13). (Fig 1 and Table 1)

**Table 1:** Geographical distribution of the isolates recovered from Veracruz, Mexico.

Jurisdictions	MR	PR	MDR	XDR	Total
**North Zone (N)**					
1	0	1	2	0	3(2.68%)
2	0	0	1	0	1 (0.89%)
3	5	1	9	0	15 (13.39%)
4	2	1	1	0	4 (3.57%)
				**Total**	**23 (20.53%)**
**Central Zone (C)**					
5	4	2	7	1	14 (12.5%)
6	3	2	9	0	14 (12.5%)
7	1	0	0	0	1 (0.89%)
8	11	7	29	0	47 (41.96%)
				**Total**	**76 (67.86%)**
**South Zone (S)**					
9	1	3	0	0	4 (3.57%)
10	1	1	0	0	2 (1.78%)
11	3	0	4	0	7 (6.25%)
				**Total**	**13 (11.6%)**
				**Total**	**112 (100%)**

### Description of the population spoligotype structure of the DR and MDR-TB strains

**Fig 2:**
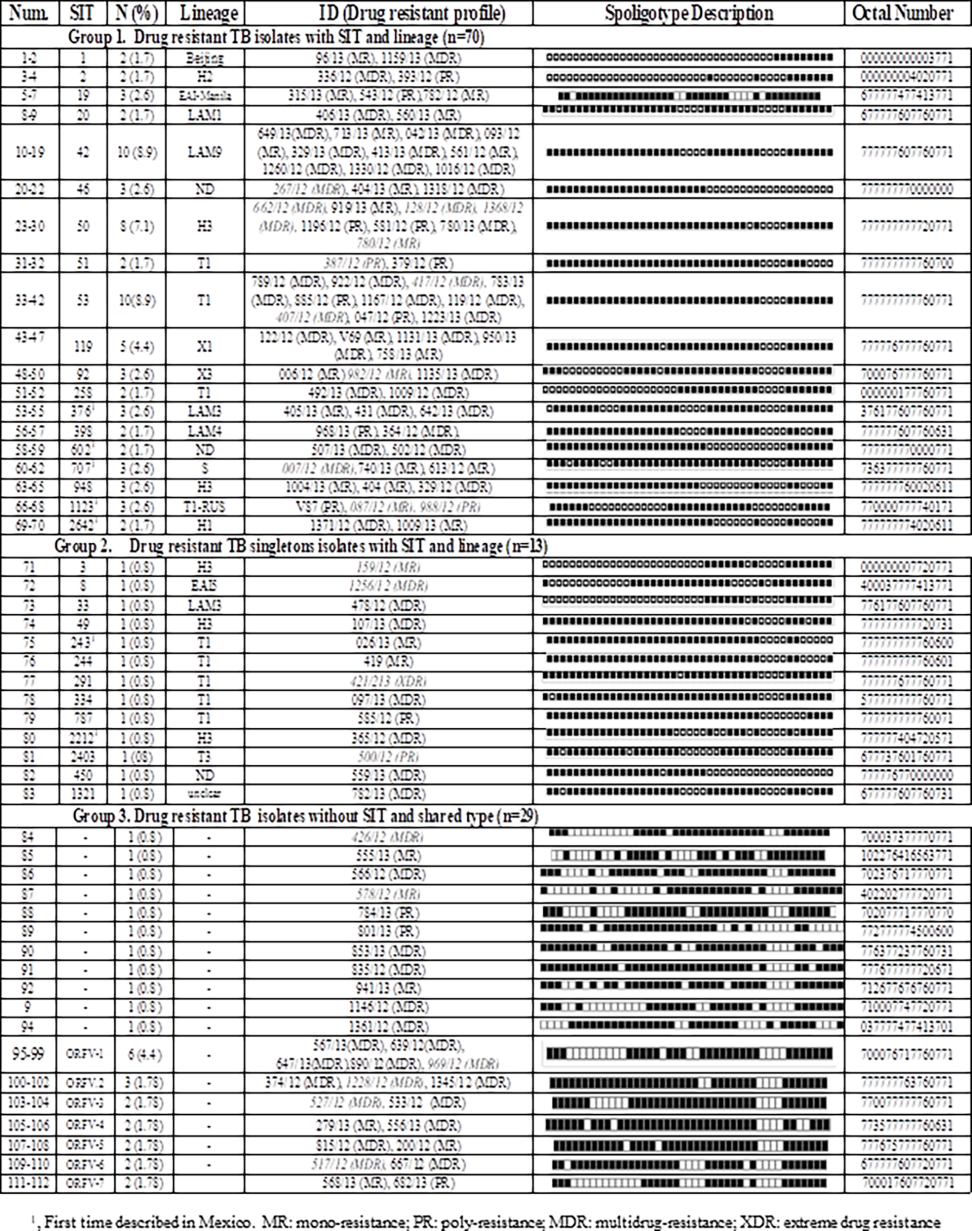
Groups according to SIT/lineage and cluster formation on drug (DR) and multidrug-resistant (MDR) TB isolates from Veracruz, Mexico.

Of the 112 resistant 83 (74%) isolates were classified in 34 (66%) recognized SITS included in eight major lineages, predominated by the “Euro-American” global lineage 4 (69%). Considering the spoligotype, SIT/lineage and cluster formation, the isolates were classified in three major groups (Fig 2). The first group included 70 (63%) isolates with 19 SITs/lineages, forming the same number of clusters. The second group included 13 (12%) isolates with the same number of SITs but without formation of any clusters (singletons). The third and last group included 29 (25%) isolates with not SIT/lineage assignation (orphans) and with seven clusters including 19 isolates.

The most frequent lineage was T1, which was found in 27 (24%) isolates and included three sub-lineages (T1, T3 and T1-RUS). The lineage LAM was detected in 18 (16%) strains, including four sub-lineages (LAM1, LAM3, LAM4 and LAM9). The Haarlem (H) lineage was observed in 17 (15%) isolates and included three sub-lineages (H1, H2 and H3). Less frequently found lineages were X, observed in eight (7%) isolates, classed into two sub linages (X1 and X3). At a lower proportion, the East Asia India-Manila (EAI-manila) and S lineages were observed in three (2.6%) isolates each, and the Beijing and EAI5 lineages were observed in two (1.7) and one (0.8%) isolates, respectively. Almost all these lineages were observed in the different geographical zones throughout state (Fig 1 and Fig 2).

The most abundant SITS were SIT53 (T1) and SIT42 (LAM9), with 10 isolates each, and SIT50 (H3) with eight isolates. These were followed by SIT 119 (X1) with five isolates, and then the SIT19 (EAI-Manila), SIT46 (ND), SIT92 (X3), SIT 376 (LAM3), SIT707 (S), SIT948 (H3) and SIT1123 (T1-RUS), with three strains each (Fig 2). The SIT1 (Beijing), SIT2 (H2), SIT 20 (LAM1), SIT51 (T1), SIT258 (T1), SIT398 (LAM4), SIT602 (ND), SIT 2642 (H1) were observed with two isolates each (Fig 2). Is important to mention that in the third group 18 (16%) orphan isolates formed seven clusters: ORF1-V with five isolates (700076717760771), ORF2-V (777777763760771) with three, and ORF-V3 (770077777760771), ORF-V4 (773577777760631), ORF-V5 (777675777760771), ORF-V6 (677777607720771) and ORF-V7 (700017607720771) with two isolates each (Fig 2).

### MIRU-VNTR analysis and identification of clonal complexes

The results of the MIRU-VNTR 24 loci analysis showed that the 31 isolates were classified into ten clonal complexes, which corresponded to the following lineages by spoligotyping: CC1-T1-1 (n = 8, 293245433335336204344a62), CC2-LAM9 (n = 5, 25221643242411615 4403933); CC3-X1 (n = 3, 253234442334525153344043), CC4-H3-1 (n = 3, 223236333734 425154343843), CC5-ORFV-1 (n = 2, 263244342335535256544ª44), CC6-X3 (n = 2, 00023 0030420205100000004), CC7-ORFV-5 (n = 2, 234234523425236235444623), CC8-ORFV-6 (n = 2, 244216330334536264443643), CC9-EAI (n = 2, 214225432353276224352623) and CC10-46 (n = 2, 364246523246525235544553).

The global HDGI calculated was 0.99, and the individual diversity index showed that only one allele was poorly discriminant, Miru04 (0.18). Two were moderately discriminant Miru2 (0.5), Miru24 (0.53). While the remaining 21 were highly discriminant: Miru20 (0.62), ETRA (0.63), Mtub 34 (0.63), Mtub 21 (0.64), ETRC (0.66), ETRB (0.66), Miru23 (0.67), Mtub 29 (0.67), Miru 31(0.68), Mtub 39 (0.69), Miru 39 (0.70), Miru26 (0.71), Miru27 (0.71), Miru16 (0.76), Miru 40 (0.79), Miru10 (0.79), Mtub 30 (0.82), Mtub04 (0.83), QUB11B (0.82), QUB4156 (0.83) and QUB 26 (0.88).

### Cluster formation and drug resistance characteristics

**Fig 3:**
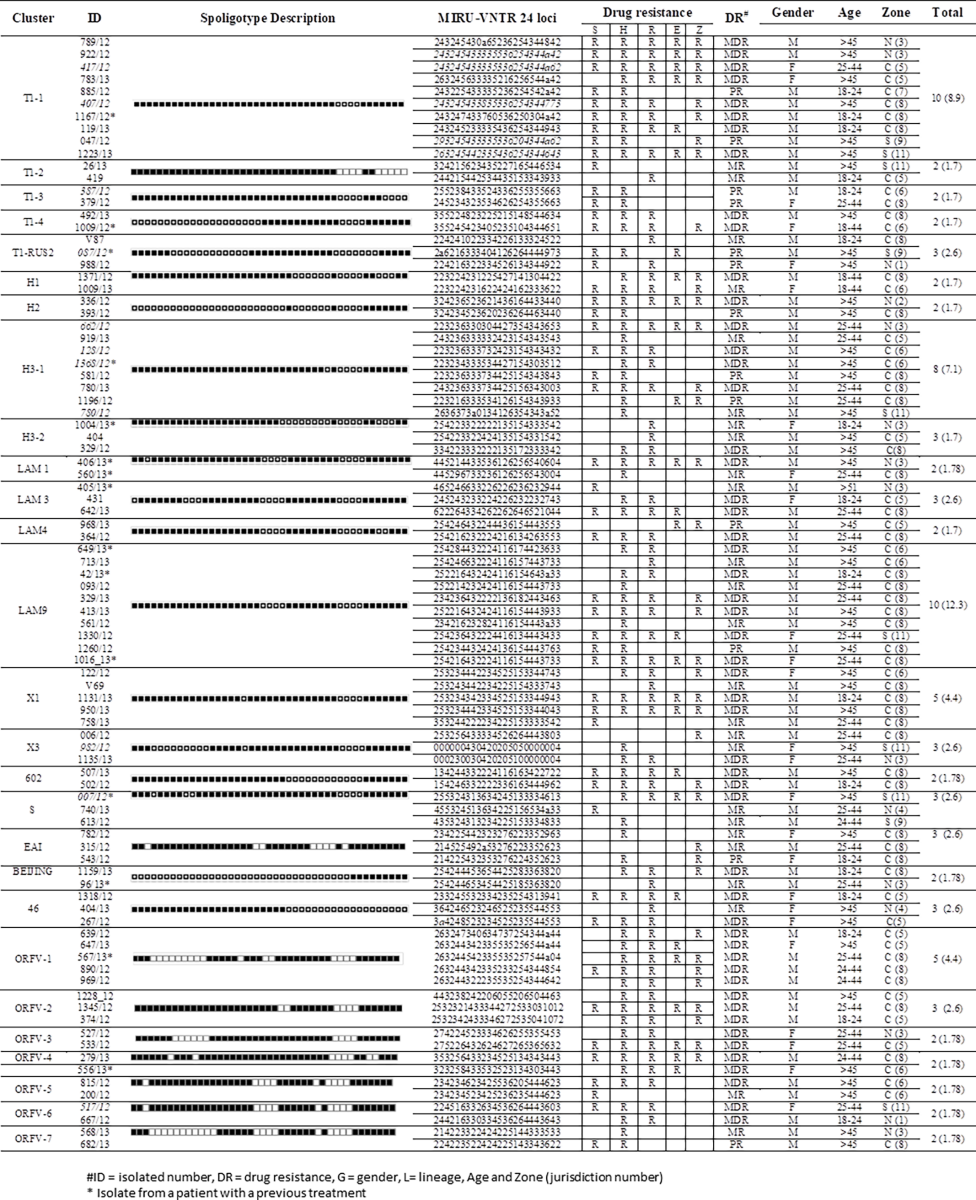
Characteristics of clusters observed by the UPGMA algorithm in isolates from Veracruz, Mexico.

Three clusters were the most abundant (Fig 3); (1) cluster T1-1, including ten isolates, eight of them were MDR, and distributed throughout the state but with predominance in the CZ, and in males older than 45 years of age. (2) Cluster LAM9, consisted of ten isolates, six of which were MDR, nine were from the CZ and eight observed in males. (3) Cluster H3-1, including eight isolates, four of which were MDR, eight were from the CZ and found in male patients.

Two clusters featured five isolates each: the first was X1, and included three MDR isolates, all of which were from the CZ and predominantly from males; the second was ORFV-1, formed by orphan isolates, all of which were MDR, from the CZ and were found predominantly in males. (Fig 3)

Eight clusters had three isolates: T1-Rus2, H3-2, LAM3, X3, S, EAI, 46 and ORFV-3. Finally, sixteen clusters were found with two isolates: T1-2, T1-3, T1-4, H1, H2, LAM1, LAM3, LAM4, LAM, 602, Beijing, ORFV-2, ORFV-4, ORFV-5, ORFV-6, and ORFV-7 (Fig 3).

**Fig 4:**
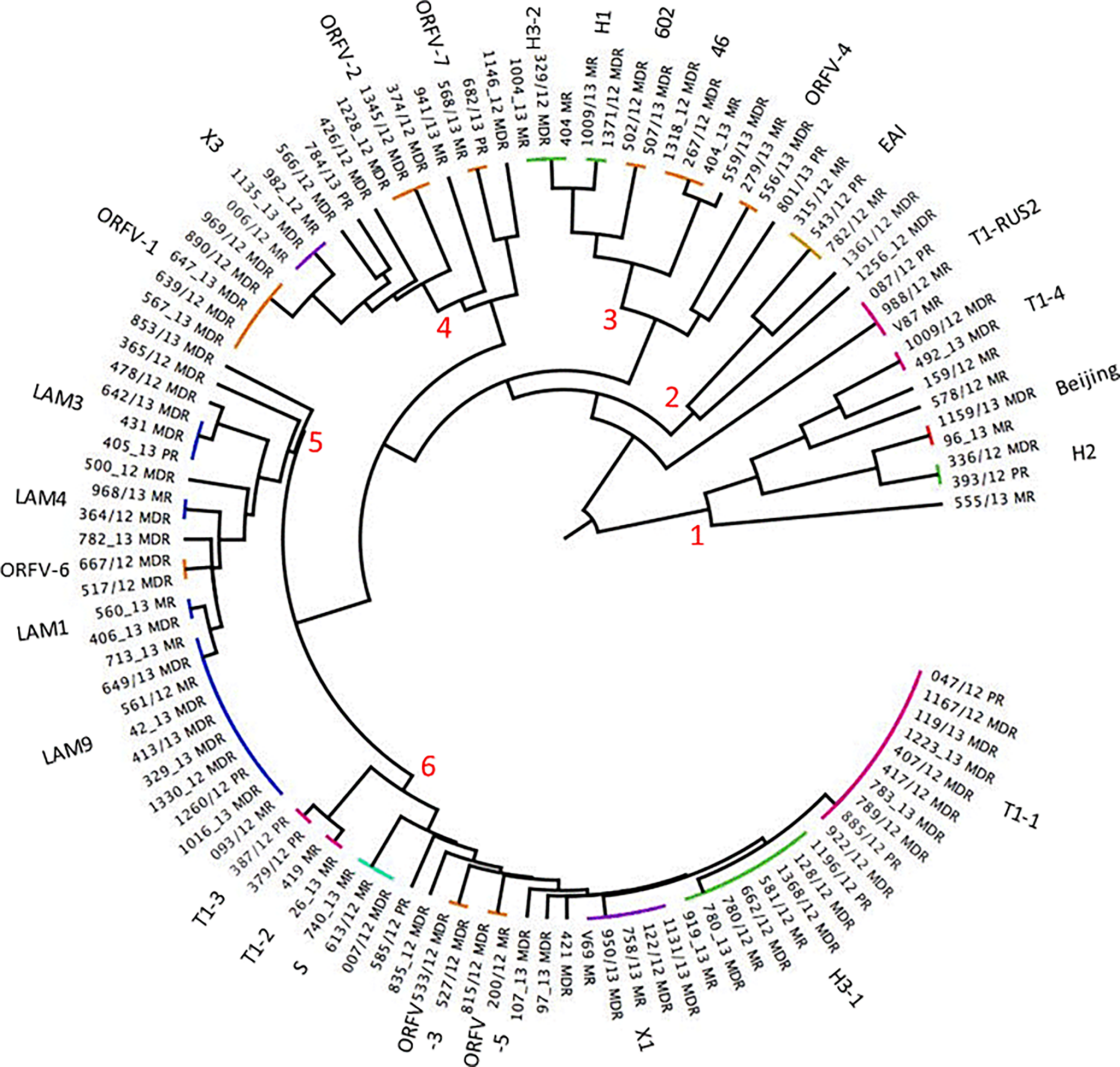
Tree polar arrangement dendogram, from DR and MDR isolates of Veracruz, Mexico.

Of the 91 isolates from patients with a first-time diagnosis 75 formed 26 clusters and the rest were singletons. Only 16 cluster (T1-2, T1-3, H1, H1, H2, LAM4, X1, X3,602, EAI, 46, ORFV-2, ORFV-3, ORFV-5, ORFV6 and ORFV-7), were conformed exclusively by individuals with a first time diagnostic and only four (602, ORFV-2, ORFV-3, and ORFV6) included isolates that were exclusively multidrug resistant. Meanwhile, of the 21 isolates from individuals with retreatment, 15 were observed in twelve clusters of which only one (LAM3), consisted of two individuals with a retreatment, but with different patterns of resistance (Fig 3).The tree polar arrangement dendogram shows the presence of 6 major groups (Fig 4). The first include three clusters with the H2, Beijing and T1-4 lineages. The second group includes only one cluster with isolates bearing the EAI lineage. The third group included five clusters, two with H-lineage (H1-H3-2), one orphan (ORFV-4) and two without lineage (602 and 46). The fourth included four clusters, only one was X3, and the remaining were orphan (ORFV-1, ORFV-2, ORFV-7). The fifth group considered four clusters with LAM sub-lineages (LAM1, LAM3, LAM4, LAM9) and one orphan cluster (ORFV6). The last group was the most abundant, and considered 8 clusters, three with T1 lineage (T1-1, T1-2 and T1-3), and remaining were S, ORFV-3, ORFV-5, X1 and H3-1.

### Association of variables with clusters, SIT and lineages

The analysis of association between variables only showed that isolates and clusters with an orphan genotype had 2.5 (IC 1.05–5.86) and 3.3 times more risk (IC 1–11.03) of having a MDR-TB phenotype, respectively.

**Fig 5:**
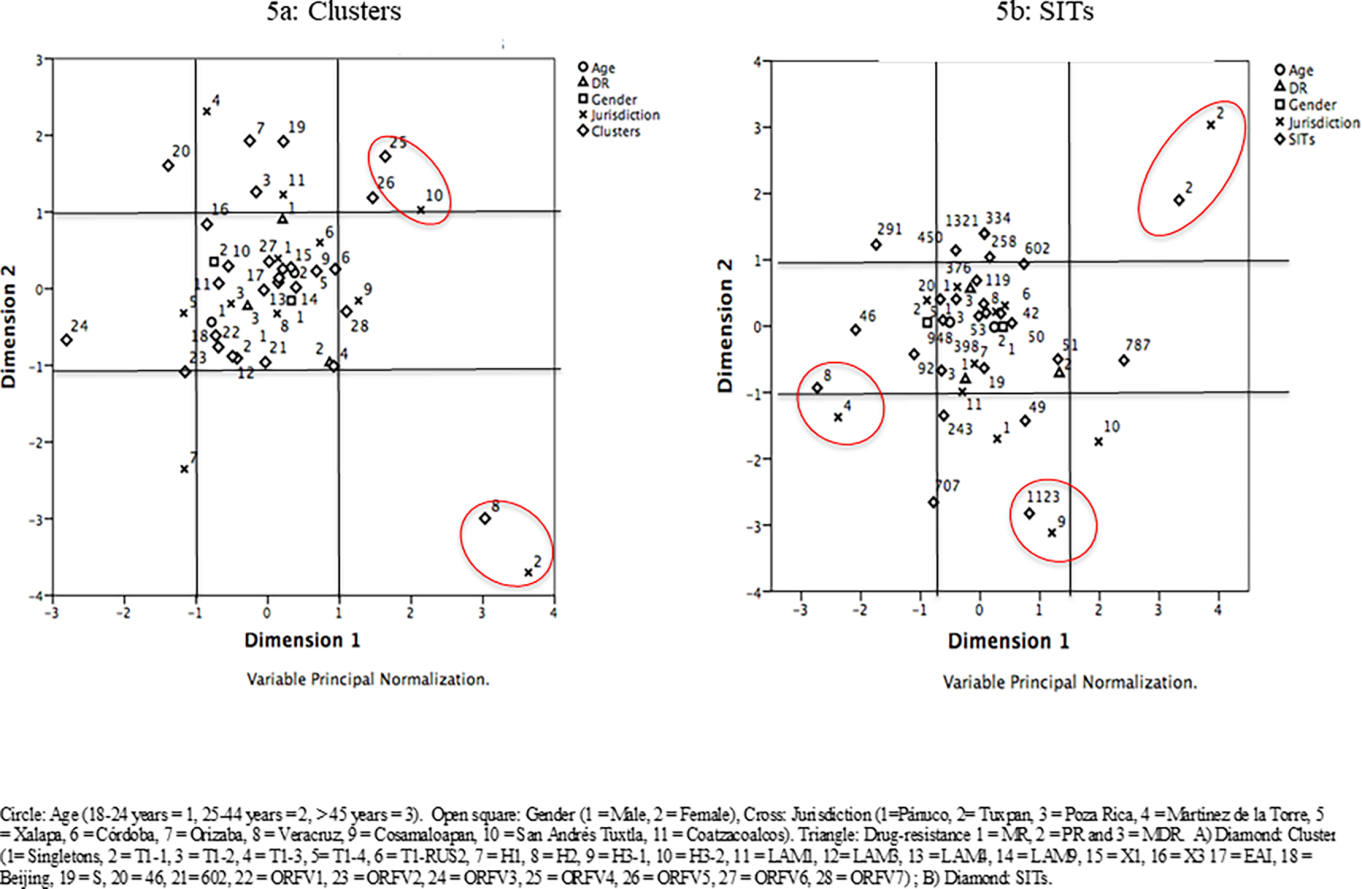
Graph of points by category of multiple correspondence analyses of the variables, age, jurisdiction, resistance profile, gender and; a) Clusters and b) SITs of the 112 isolates of drug-resistant TB analyzed.

Through multiple correspondence analyses, and considering cluster and SIT, it was possible to observe some associations within regions of the state of Veracruz. The cluster ORVF4 showed an association with jurisdiction 10 San Andres Tuxtla, (Diamond 25 with cross 10, Fig 5A) (inertia values ACS Cluster *vs*. Jurisdiction; 0.268 and 0.255, respectively), while the cluster H2 had an association with jurisdiction 2 Tuxpan (Diamond 8 with cross 2, Fig 5A) (inertia values ACS Cluster *vs*. Jurisdiction 0.493 and 0.491, respectively). Considering the SIT as an element of aggrupation, SIT8 (EAI5) showed association with the jurisdiction 4 Martinez de la Torre (NZ) (Diamond 8 with cross 4, Fig 5B) (inertia values ACS SIT *vs*. Jurisdiction, 0.241 and 0.388, respectively). The SIT 1123 (T1-Rus) and jurisdiction 9 Cosamaloapan (Diamond 1123 with cross 9, Fig 5B) (inertia values ACS SIT *vs*. Jurisdiction, 0.203 and 0.229, respectively) were associated. Finally, SIT2 (H2) and jurisdiction 2 Tuxpan (NZ) had a significant relationship (Diamond 2 with cross 2, Fig 5B) (inertia values ACS SIT *vs*. Jurisdiction, 0.493 and 0.491, respectively).

## Discussion

From the 20,000 cases of TB reported annually in Mexico, between 1,200 and 1,500 are DR. The state of Veracruz contributes 11% (2,000) of the total number of TB cases, of which 8% (150) are DR. In addition, Veracruz also occupies the first place in terms of the number of prevalent cases of MDR-TB in the country. For these reasons, the state is placed as one of the most significant contributors to the DR-TB and MDR-TB problem in Mexico [31]. These data help to explain why 55% (62) of the isolates analyzed in this study were MDR, as well as why the sample analyzed almost corresponded to all of the cases diagnosed as DR-TB in one year in the state.

The 82% of the isolates (75/91) from patients diagnosed as a new case were located in a cluster. Lineages T and H had some tendency to include these new cases, but with different patterns of resistance. However, in the seven clusters containing the 18 isolates classified as orphans, 16 were new cases and thirteen were found to be multidrug-resistant. It will be necessary to further characterize these orphans isolates, using additional molecular markers to establish their belonging to a specific lineage [32], as well as the factors that promote their tendency to develop drug resistance. On the other hand, 71% (15/21) of the isolates from a pre-treatment Tb patient were located in clusters. Clusters with a LAM lineage included 28% (6/21) of these individuals, of which only three were multidrug-resistant. This information show that the DR-TB in the region seems to be mainly dominated by clones belonging to diverse lineages with diverse resistance profiles and wide dispersion. The information will be of great help to suggest improvements to the surveillance of DR-TB, and prevent its spread.

Resistance to isoniazid was the most frequent; it was observed in 71% (80/112) of the recovered isolates. Of these, 27% (31/112) and 50% (9/112) were isolated with a mono- and poly-drug resistant character. It has been recently suggested that resistance to isoniazid arises prior to the appearance of rifampicin resistance [33], therefore 35% (40/112) of the isolates studied could evolve to develop resistance to rifampicin and to be multidrug-resistant. As a consequence, it is necessary to develop a closer surveillance system in the state, with a particular emphasis on isolates where resistance to isoniazid is observed and with the aim to reduce the evolution of DR to MDR and subsequent dispersion within the population.

Analysis of the 112 recovered isolates allowed us to establish the first detailed description of the genetic structure of one of the most abundant collections of DR-TB and MDR-TB isolates from southeast Mexico. The results showed that 69% of the isolates were located within the "Euro-American" global lineage 4, which includes the T, LAM and H lineages [34,35], while 25% of the strains were found as orphans, with the remaining 6% in two Asian lineages.

The predominant nature of lineage 4 and the low presence of lineages 2 and 3 (Beijing and EAI-manila) support previous reports from DR and MDR-TB isolates of central and northern Mexico, as well as from other Latin American countries (Table 2). The T-lineage showed the highest frequency, coinciding with previous reports from Mexican isolates; however, in South American countries, the LAM lineage seems to be predominant (Table 2). From these observations, two questions emerge: how could the socio-demographic, geographic and genetic factors of the Latin American population could be promoting the dispersion of members of Lineage 4 over 2 and 3 [36], and how could this factors also could influence the major occurrence of T over the LAM lineage in Mexico. Further molecular epidemiology studies are necessary to answer these questions.

**Table 2:** Studies reporting distribution of lineages in *M*. *tuberculosis* drug and multidrug resistant isolates in Latin America.

Country and Period	No. of strains /type	H	T	LAM	S	Beijing	X	EAI	Orphans or unclassified	Reference
Argentina, 2003–2009	787/MDR	36.3%	13.9%	**38.8%**	2.8%	1.5%	0.9%	-	3.8%	[37]
Argentina, Brazil, Chile, Colombia, Venezuela, 2004–2008	951/MDR	28.9%	17.4%	**37.2%**	-	1.3%	-	-	-	[38]
Brazil 2015	104/DR-MDR	5.7%	14%	**66%**	1.9%	1.9%	-	-	8.6%	[39]
Brazil 2012	121/MDR	4%	22%	**57%**	-	-	0.8%	-	-	[40]
Brazil 2014	99/MDR	12%	17%	**47%**	-	-	6%	1%	-	[41]
Peru 2015	209/DR,MDR	13%	8.6%	**59%**	-	8%	4%	-	-	[42]
Peru 2014	142/XDR	**43.6%**	27.4%	16%	-	9%	1.4%	-	-	[43]
Colombia 2011	76/DR,MDR	22.5%	5.6%	**30%**	-	15%	3.8%	-	23.1%	[44]
Mexico 2013	109/MDR	3.6%	**56%**	4.6%	-	2.75%	0.9%	4.6%	20.1%	[18]
Mexico 2002–2013	1237/Sensitive /DR	3.1%	**20.3%**	6.8%	1.29%	0.72%	11.4%	6.8%	-	[19]
Mexico 2017	112/MDR	15%	**24%**	16%	2.6%	1.5%	7%	2.6%	25%	This study

We identified 32 SITs, of which 17 (53%) (1, 2, 19, 20, 42, 46, 49, 50, 51, 53, 92, 119, 291, 334, 948, 787 and 2212) had been previously described in DR-TB and MDR-TB isolates circulating in Mexico [18,19], and other Latin American countries [40,41,43,44].

Given their relationship with high virulence and development of drug resistance, seven SITs draw attention: SIT1/Beijing and SIT19/IIA-Manila, forming two clusters each with three isolates, of which only one was MDR. The W-Beijing lineage is considered important because of its predominance in Asian countries, high infectiousness and tendency to develop multidrug resistance[45–47].The occurrence of these lineages in the area confirms their extensive distribution in Mexico, as well as their apparently limited participation in the generation of multidrug resistance [15,18,19,48].

The SIT20/LAM1 was observed in two isolates forming a cluster. This SIT has special importance since it specifically includes isolates with the RDRio genotype, which is characterized by being highly virulent and a more efficiently causing infection [49]. Interestingly, one of the isolates carrying this SIT showed resistance to all first-line drugs. Ten isolates presented the SIT42/LAM9 and two isolates presented the SIT398/LAM4, of which nine were MDR. It has been described that both SITs present a combination of missing and bearing the RDRio genotype. The SIT376/LAM3 formed a cluster with three isolates, two of which were MDR. However, the RDRio genotype is absent in the members of this SIT[49,50]. Up to date, there are no reports confirming either presence or absence of the RDRio genotypic marker in isolates with LAM lineage circulating in Mexico. It will therefore be necessary to confirm the occurrence of this genotype as well as determine its influence on the development of drug- and multidrug-resistant TB.

SIT50/H3 has a high frequency of *katGS315T* mutation in isoniazid resistant TB strains[51] and DNA repair genes that allow greater adaptability to hostile environments [52]. All eight isolates with the SIT50/H3 identified here were also resistant to isoniazid. This SIT has been described in Mexico [18,19]; nevertheless, descriptions of mutations explaining DR or MDR character in isolates with this lineage are scarce [20,53]. We have planned to perform additional analyses to confirm the presence of mutations in the *katG* gene in SIT50 and the remaining SITs of the H lineage found in the setting, such as SIT2/H2, SIT948/H3, SIT2642/H1, in order to evaluate their influence on generation, dispersion and resistance to isoniazid.

Fifteen isolates (13%) were located within seven SITs described for the first time in Mexico (243, 376, 602, 707, 1123, 2212 and 2642). According to the SITVIT-Web database [26], two marked behaviors were observed in terms of geographical location of these SITs. Four SITs has been reported only in countries from the American continent: SIT376/LAM3 observed in Brazil, Venezuela and Guatemala; SIT707/S described in Argentina; SIT2642/H1 in Paraguay and SIT2212/H3 detected in the United States. The second group included isolates with a wider distribution, including SIT243/T1 described in Belgium, Italy, Zambia and United States; SIT1123/T1-RUS, detected in Poland, Belgium, Bangladesh, Turkey and the United States. The report of this SIT by our study represents its first description in a Latin American country. Finally, SIT602 was observed in Germany, Turkey, Spain, Brazil, Germany United States, France and South Africa. This SIT included two MDR-TB isolates in a cluster and the spoligotype matched with a TB-MDR isolate recovered in 2003 from the state of Tabasco (south of Mexico) and classified as orphan [18]. This information shows that this SIT seems to have been circulating in Mexico for more than 10 years and with a strong tendency to develop and/or transmit a MDR phenotype.

The significant numbers of spoligotypes (52), lineages (8), clusters (27), clonal complexes (10) and orphaned spoligotype patterns (29) identified demonstrates the high and important genetic diversity of DR-TB and MDR-TB isolates circulating in the area. Transmission rate was calculated at 56%, showing that around half of the TB-DR isolates were generated from direct transmission while the remainder originated from different sources. In this sense, all of the individuals included in the study were originated from the state of Veracruz and stated that no trips outside Mexico had been undertaken in the last three years. It is important to mention that the state of Veracruz has an important flow of Central and South American immigrants were the final destination is the United States and also remark that the state has three of the five major seaports of Mexico. These factors may explain the possible importation and subsequent distribution of some of the genotypes circulating in the area, a similar scenario has been described in the state of Baja California, a state located in the north of Mexico [48].

The high diversity of the genotypes/lineages found in our work, especially from Lineage 4, seems to be a constant in Latin American countries (Table 2); however, this differs to that seen in Asian, European or African countries, in which genotypes/linages from groups 1, 2 and 3 or from a more specific Lineage (e.g., Beijing) are those mainly responsible for the transmission and dispersion of DR-TB and MDR-TB isolates [54–57]. This could be accounted by the founder effect observed in MDR isolates from Peru [42], and Ukraine [58] where the predominance of specific lineages seems to confine the dispersion of new o less frequent lineages in the same geographic region.

According to the risk analysis, having only an orphan genotype or being within a cluster with this characteristic (ORFV) was the only variable that showed an associated risk of developing MDR-TB (OR 2.5 and OR 3.3, respectively). However, the multiple correspondence analyses showed relatively high values of inertia (associations) for certain clusters and specific SITs with jurisdictions, evidencing an apparent initial development of outbreaks of MDR-TB isolates in certain geographic areas. However, to confirm these risks, it will be necessary to perform additional genotypic analysis including sensitive TB isolates, considering that in a heterogeneous population the probability of an MDR-TB case being observed within one specific cluster decreases on the contrary the sensitive cases increase. Perhaps this statistical tool could be useful in future studies where an evaluation of numerous variables and identification of possible associations, considering data with significant disaggregation would be performed.

One of the most important limitations of our study was the impossibility of performing drug sensitivity tests at time of initial TB diagnosis. In Mexico, drug susceptibility tests are only performed when a positive bacilloscopy is observed at the second or third month of treatment. It was therefore not possible to determine whether the individuals carrying certain SITs had a primary or secondary type of resistance, and it was consequently impossible to determine in detail the tendency or capacity of certain SITs to develop or transmit DR or MDR.

A second limitation of our study was related with the absent of genotypic information about drug sensitive TB isolates, all this information could help us to establish a more correct proportion of drug resistance isolates in each cluster/clade, lost or bias in the data and potential risk factors. Nevertheless, the results obtained in this study show predominance tendencies of some lineages related with drug resistant that should be confirmed with detail further, also this study indicate the urgent need to implement these genotyping tools within a system of epidemiological-molecular TB surveillance in Mexico.

In conclusion, our study illustrates the utility of spoligotyping and MIRU-VNTRs for establishing an initial baseline of the genotypic lineages of DR-TB and MDR-TB strains currently in circulation in Veracruz. The results highlight an important diversity of circulating genotypes, with a significant proportion of T lineages, followed by LAM and H. Further studies are required in order to better elucidate the genetic structure of the DR-TB and MDR-TB lineages circulating in Mexico; this information will help us to determine how these lineages could be influenced by geographic, ethnic or migration factors, and also assess the effectiveness of the TB control program, with an emphasis on surveillance of drug resistance.
